# Using Calcination Remediation to Stabilize Heavy Metals and Simultaneously Remove Polycyclic Aromatic Hydrocarbons in Soil

**DOI:** 10.3390/ijerph15081731

**Published:** 2018-08-13

**Authors:** Peixin Wang, Xiaojie Hu, Qianjia He, Michael Gatheru Waigi, Jian Wang, Wanting Ling

**Affiliations:** Institute of Organic Contaminant Control and Soil Remediation, College of Resources and Environmental Sciences, Nanjing Agricultural University, Nanjing 210095, China; 2017103049@njau.edu.cn (P.W.); 2014203012@njau.edu.cn (X.H.); soilchem@njau.edu.cn (Q.H.); mike2013103193@163.com (M.G.W.); wj308119@sina.com (J.W.)

**Keywords:** soil remediation, calcination, heavy metals, polycyclic aromatic hydrocarbons, co-contamination

## Abstract

Co-contaminated soils containing heavy metals and polycyclic aromatic hydrocarbons (PAHs) are an environmental and human health risk. Research into the remediation of these soils is imperative. In this paper, a novel investigation utilizing calcination technique to stabilize heavy metals and simultaneously remove PAHs in soil was conducted. Calcination temperature (300–700 °C) was observed to play a dominant role in heavy metal stabilization and PAH removal in soils. However, calcination time (0.5–8 h) had no significant effect on these contaminants during calcination at different temperatures. Considering the remediation cycle requirements and economic costs of engineering, we suggested that the optimal calcination condition for Zn, Cu, naphthalene, and fluoranthene was at 700 °C for 0.5 h, and the corresponding stabilization or removal efficiency values were 96.95%, 98.41%, 98.49%, and 98.04%, respectively. Results indicate that calcination as a remedial strategy exhibits a bright future for practical applications in the simultaneous stabilization of heavy metals and PAH removal from co-contaminated sites.

## 1. Introduction

With the accelerated process of urbanization and transfer of industries in China, a general trend is being observed in the relocation of industrial enterprises to economically developed or fast-developing regions. At the same time, a large number of diverse and complex contaminated sites have been abandoned. Soil pollutants in these sites are not only of serious concern to the ecosystem and agricultural production, but also to human beings. Such sites hinder city construction and local economic development. The contamination in most of these sites is no longer limited to individual pollutants. The most common types of co-contamination present in these soils are combined heavy metal–heavy metal, organic–organic, and heavy metal–organic contamination [1]. Heavy metals and polycyclic aromatic hydrocarbons (PAHs) are the two main pollutants that are typical representatives of combined heavy metal–organic pollution found in contaminated soils. As such, there is considerably increased complexity in remediating such contaminated soils due to their elusive, long-term, and irreversible properties in the soil environment. Therefore, it is imperative to develop effective and feasible remediation technology for such co-contaminated sites. 

At present, various studies have reported on the remediation technology of soils contaminated by heavy metals and PAHs. Summarized from existing literature, remediation technologies that can address both heavy metals and PAHs in contaminated soils mainly include soil washing [2], supercritical fluid extraction [3], and electrokinetic technology and bioremediation [4,5,6,7]. In the last decades, phytoremediation and microbial bioremediation have received extensive attention due to their low cost and wide adaptability with no secondary pollution. However, these techniques are generally time-consuming, with difficult remediation in heavily polluted soils, and mostly focus on agricultural soils. Calcination treatment is considered as a commonly used physical remediation with the advantages of high efficiency, simplicity, and rapidity. In addition, it is suitable for site remediation projects that respond to emergent environmental accidents and short construction time requirements. Recent studies show that this treatment is very effectual in removing Hg from chlor-alkali industry wastes while obtaining mercury removal efficiency close to 100% [8,9,10]. Leachability according to the US Environmental Protection Agency (EPA) Toxicity Characteristic Leaching Procedure (TCLP) leaching test decreased below the threshold value of 0.2 mg Hg/L after treatment at a temperature of 400 °C or higher (retorting time of 1 h). Thermally enhanced remediation of trichloroethylene (TCE) from a 50-cm-deep silty soil was demonstrated in a two-dimensional laboratory tank by Heron et al. [11]. When subjected to 37 days of heating, there was a reduction in mass of TCE in the soil from 35 to 0.072 g, corresponding to 99.8% mass removal. Nevertheless, whether this technology may also be used in the simultaneous detoxification of heavy metals and PAHs in soil still requires further research. So far, limited work has been conducted to assess critical information on calcination treatment to remediate co-contaminated soils.

To this end, the present study aimed to clarify the feasibility and performance of calcination technique to stabilize heavy metals and simultaneously remove PAHs in the soil. Batch calcination experiments were conducted at different temperatures and times. Then, the stabilization efficiency of zinc and copper (as heavy metal representatives), along with the removal efficiency of naphthalene and fluoranthene (as PAH representatives), was accordingly calculated and analyzed. The results of this investigation will fill in the blanks of the calcination remediation research, and provide new ideas for the innovation of remediation technologies of soils co-contaminated with heavy metals and PAHs.

## 2. Materials and Methods

### 2.1. Chemicals

CuCl_2_·2H_2_O, ZnCl_2_, and EDTA-2Na were all purchased from Sinopharm Chemical Reagent Co., China. Their respective molecular weights are 170.48, 136.30, and 372.24 g/mol respectively. Naphthalene and fluoranthene were provided by Shanghai Macklin Biochemical Co. from China with a purity >98%. These two PAHs were commonly found with the highest concentrations among the 16 USA EPA priority PAHs in contaminated sites. Their general physicochemical properties are given in Table 1. 

### 2.2. Contaminated Soils

Natural surface soil samples were yellow-brown soil collected from the Tanabe soil in the suburbs of Nanjing in Jiangsu Province, China, with a pH of 6.43, 15.1 g/kg soil organic carbon content, 26.3% clay, 14.1% sand, and 59.6% silt. The samples were naturally dried, ground, and sieved through a 20 mesh. Subsequently, 2 kg of the obtained soil were accurately weighed, followed by the addition of a solution of CuCl_2_·2H_2_O (5.33 g) and ZnCl_2_ (4.19 g). Then, a known amount (1.00 g for each PAH) of naphthalene and fluoranthene was added to the above mixture. Then, the soils were mixed thoroughly, aged for 40 days, freeze-dried, and stored at −25 °C for later use. After analysis, the final determined available contents of zinc and copper in the soil after contamination were 808.73 and 770.19 mg/kg, and the final concentrations of naphthalene and fluoranthene reached concentrations of 61.66 and 320.03 mg/kg, respectively. 

### 2.3. Calcination Experiments

Batch calcination experiments were conducted to determine the influence of different temperatures and time on the soil samples. Then, 8.00 g of contaminated soil were weighed into the crucible and placed in a box-type resistance furnace at a preset temperature, where the temperatures were set to 300, 400, 500, 600, and 700 °C, respectively and the calcination times were 0.5, 1, 2, 4, and 8 h. Each treatment set three parallels. After the calcination, the soils were taken out and allowed to cool naturally to room temperature before analysis. The same soil samples placed at the room temperature (20–25 °C) were used as controls, and on comparison with the initial soils no differences were observed in the concentrations of PAHs and available metals in the control soils.

### 2.4. Analysis of Available Contents of Heavy Metals in Soils

For this, 10 mL of 0.05 mol/L EDTA-2Na was added to a plastic centrifuge tube containing 2.00 g of accurately weighed soil sample. The tube was shaken in the dark for 1.5 h on a gyratory shaker to reach the equilibrium state. The solution and soil were separated by centrifugation at 4000 r/min for 5 min. Then, 0.25 mL of the supernatant was pipetted before diluting to 5 mL. The determination of Zn and Cu in soil extracts was performed using inductively-coupled plasma mass spectrometry (ICP-OES) [12]. Then, the available contents of Zn and Cu in the soil were obtained. The stabilization efficiency (S, %) of heavy metals in contaminated soils was calculated according to Equation (1) as follows:

(1) $$S=\frac{C_{i-M}-C_{T-M}}{C_{i-M}}\times 100\%$$

where $C_{i-M}$ denotes the initial available contents of heavy metals, and $C_{T-M}$ refers to the temporary available contents.

### 2.5. Analysis of PAHs in Soils

A soil sample of 2 g, which was weighed into a 25-mL glass centrifuge tube, was used to extract PAHs using an ultrasound bath with 10 mL of dichloromethane for 1 h. The solution and soil were separated by centrifugation at 4000 r/min for 10 min. Then, 3 mL of the obtained soil extracts were filtrated through a silica gel column before elution with 11 mL of a mixture of n-hexane and methylene chloride (volume ratio of 1:1). The eluent was collected in a rotary evaporation bottle, concentrated to dryness at a constant temperature of 40 °C, before methanol was added to a final volume of 2 mL. The final soil extracts were then analyzed by high-performance liquid chromatography (HPLC) after filtration through 0.22-μm syringe filters to obtain the PAH concentrations in soil [13]. The removal efficiency (R, %) of PAHs in contaminated soils was calculated according to the following equation:

(2) $$R=\frac{C_{i-P}-C_{T-P}}{C_{i-P}}\times 100\%$$

where $C_{i-P}$ and $C_{T-P}$ are the initial and temporary concentrations of PAHs in soils, respectively.

### 2.6. Statistical Analysis

All data were processed using Microsoft Excel 2010 (Microsoft, Redmond, WA, USA). Data in figures and tables represent mean values. The standard deviation among parallel samples is shown in the figures as error bars. Data were analyzed using SPSS version 11.0 (SPSS, Inc., Chicago, IL, USA). The differences among treatments were tested using ANOVA, while Tukey’s test (with a level of *p* < 0.05) was used for significance. Graphs were generated in Microcal Origin 2016 (version 9.2, Northampton, MA, USA).

## 3. Results and Discussion

### 3.1. Stabilization of Heavy Metals in Soil

**Figure 1:**
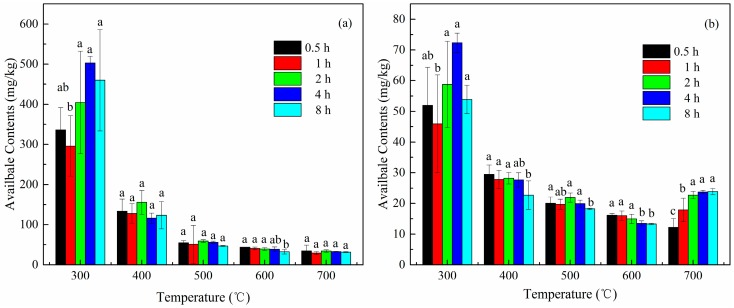
Effects of different temperatures and time on stabilization of zinc (**a**) and copper (**b**) in soils during calcination. Different letters indicate significant difference (*p* ≤ 0.05) between treatments of different calcination time at the same temperature.

The changes in the available contents of zinc in the soil with increasing calcination temperature are shown in Figure 1a. The decline in the available contents of zinc when calcinated for 4 h was the most dramatic, with the contents decreasing from 503.19 to 32.67 mg/kg. As seen, the general trend during different calcination times (0.5, 1, 2, 4, and 8 h) was roughly the same. However, as temperatures increased, the rapid decline in the beginning changed to a gradual decrease of Zn contents, to a minimum at 700 °C. The results above indicate that calcination could exert a good effect on the stabilization of zinc in soils.

The analysis of the stabilization efficiency of zinc in contaminated soils utilizing calcination treatment under different conditions further supports this result. It can be observed from Table 2 that the temperature had a more pronounced effect on the contents of zinc, in contrast to time. A rapid increase in the stabilization rate of zinc was observed from 300 to 400 °C. The stabilization efficiency for all reached over 80% at 400 °C under the set time. Then, the stabilization efficiency rose slowly as the temperature increased from 400 to 700 °C, to a maximum of 700 °C, which proved that the stabilization effect of zinc at this temperature is relatively better (up to nearly 97%). It can also be seen that there is no fixed variation in the stabilization efficiency of zinc with a prolonged calcination time.

As exhibited in Figure 1b, the available contents of copper in the soil showed a tendency of slow decline with increasing temperature. This decelerated drop led to a decrease in the contents with the drop in temperature to a minimum of 600 °C. Similar to zinc, the available copper contents showed a noticeable decline after calcination for 4 h, decreasing from 72.34 to 13.52 mg/kg, which implied that calcination treatment demonstrated a good immobilization effect on copper.

The stabilization efficiency of copper in contaminated soils utilizing calcination treatment under different conditions is provided in Table 3. All of the data exceed 90%, firmly supporting the above point of view. The stabilization efficiency of copper at 300 °C is 90.61–94.04%, reaching a relatively high level. During the temperature rise (300–600 °C), it was found that the efficiency kept rising steadily. The efficiency of stabilization subsequently showed no significant increase. Evidently, the stabilization efficiency of copper reached the highest value of 98.41% when the copper in contaminated soil was calcinated at 700 °C for 0.5 h, representing the optimal condition for copper stabilization by calcination technology. Meanwhile, the stabilization efficiency shows no significant correlation with the time of calcination.

Previous studies suggest that calcination treatment is a method for either removing some volatile heavy metals (e.g., Hg and As) from the soil or heat-stabilizing them by heating so that remediation is achieved. Wei et al. (2001) studied the mechanism of calcination stabilization of copper on minerals. Cu(OH)_2_ adsorbed or deposited on the mineral after curing from 300 to 900 °C for 1 h was converted to CuO, which is poorly soluble and difficult to elute, thus stabilizing on the surface of minerals [14]. The curing degree of heavy metals on the surface of minerals would increase with the rise in temperature. Spalding (2001) used the method of heat stabilization of radioactive elements in the soil [15]. These elements could diffuse to the internal lattice of the soil from the surface of the soil at high temperatures, thus reducing their environmental risks. The concentration and potential lability and leachability of Cr, Cu, Fe, Mn, Ni, Pb, and Zn in the native sludge and in the thermal-treated sludge samples were studied using a five-step chemical fractionation method and a column experiment [16]. As a consequence of heating for 3 h at 180 °C, 300 °C, and 400 °C, respectively, the trace metals were more strongly fixed in the treated sludge. This fact constitutes an advantage to reduce the possible release of metals in solids using heating methods. No matter which method is used to remediate soils polluted by heavy metals, the immobilization mechanisms can typically be classified into two groups. These include chemical stabilization, which entails the transformation of heavy metals into less soluble species or less toxic forms, and physical encapsulation, the condensing of heavy metals into a low permeability waste form before isolating them from the environment [17].

The finding of this work using calcination to stabilize heavy metals in contaminated soil was supported by the observations in the literature above, which reported that the stabilization efficiency of heavy metals would generally enhance with increasing calcination temperature. Internal morphological alternation which made heavy metals less soluble presumably occurred, like the conversion of amorphous hydroxides to oxides, contributing to immobilization of metals. Considering the remediation cycle requirements and economic costs of engineering, it is usually sufficient to calcinate at 600–700 °C for 0.5–1 h.

### 3.2. Removal of PAHs in Soil

**Figure 2:**
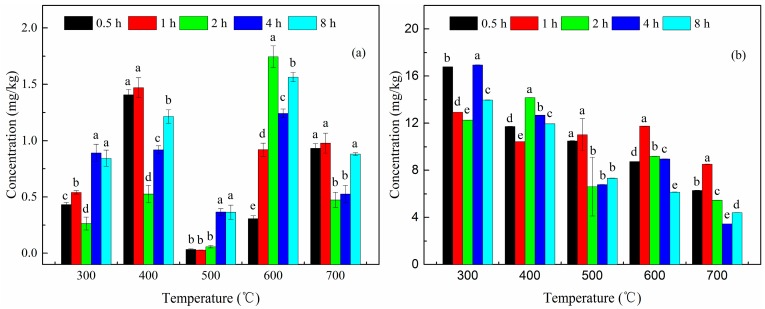
Effects of different temperatures and time on removal of naphthalene (**a**) and fluoranthene (**b**) in soils during calcination. Different letters indicate significant difference (*p* ≤ 0.05) between treatments of different calcination time at the same temperature.

The calcination treatment effectively removed PAHs from the soil based on the observations of residual concentrations and removal efficiency of PAHs in soils. As seen in Figure 2a, the naphthalene concentration changed from 1.47 (400 °C) to 0.03 mg/kg (500 °C), declining sharply when the calcination time was 1 h, thus corresponding to a high removal rate of 99.95% (Table 4). In general, the variation of naphthalene concentration in the soil displayed no significant correlation with the increase in either calcination temperature or time. 

The concentration of fluoranthene in the soil with the temperature increase exhibited a comparatively significant decline trend in Figure 2b. It decreased from 16.96 mg/kg (300 °C) to the lowest value of 3.45 mg/kg after calcination at 700 °C for 4 h, corresponding to a removal efficiency of 98.92% (Table 5). The concentration of fluoranthene was still almost independent of the calcination time. Consequently, the results obtained suggest that the PAH concentrations in contaminated soils can be effectively reduced by utilizing calcination treatment. Additionally, the removal rates of naphthalene and fluoranthene in the contaminated soil during calcination under different temperatures and time are provided in Table 4 and Table 5, which further appear to prove the exceptional impact of calcination technology. The removal rates were all higher than 94%, as observed in the two tables (Table 4 and Table 5). Among them, the removal efficiency of naphthalene was close to 100% at 500 °C.

Generally, it can be seen that the concentrations of naphthalene and fluoranthene in the soil show a rising trend with increasing calcination time. Moreover, taking the optimum time for heavy metals in soil into account, calcination of PAHs for 0.5–1 h was appropriate. Additionally, 500 °C is the optimum temperature condition for removing naphthalene in contaminated soil, while 700 °C is optimal for fluoranthene removal. Thereby, the optimized stabilization efficiency values of zinc and copper in soil at 700 °C for 0.5 h are 96.95% and 98.41%, respectively, while the removal efficiency values of naphthalene and fluoranthene under optimized conditions reach 99.94% and 98.04%, respectively. 

Calcination technology is a physicochemical separation process employing heat to either destroy or volatilize organic contaminants from waste matrices such as soil, sludge, and sediments [18]. It is an effective method for destroying PAHs in the contaminated plume using high temperatures [19] ranging between 900 and 1200 °C. In 1986, the US EPA conducted a remedial investigation of a heavily creosote-contaminated Superfund site located in Louisiana [20]. The site remedy included excavation and incineration of 142,000 tonnes-contaminated solids in a transportable incinerator. The incineration system destroyed 90% of the total PAHs in 40 months, thus proving successful. In another investigation conducted by Renoldi et al. [21], contaminated soil from a manufacturing gas plant site was treated in a laboratory-scale indirectly heated calcination desorber unit. After treatment at maximum temperatures above 450 °C, the concentrations of 16 PAHs reduced to below 0.05 mg/kg dry weight, which corresponded to a removal efficiency of 99.9%. In comparison with the above reports, the remediation performance on the PAHs using the calcination technique in this paper is satisfactory. PAHs after calcination were presumed to be volatilized or destroyed in the contaminated soil due to the high temperatures. 

## 4. Conclusions

This is the first investigation showing that utilizing calcination technique remediates soils co-contaminated by heavy metals and PAHs. Temperature played an essential role in stabilizing heavy metals and removing PAHs in soils under calcination treatment. The optimal removal temperature for naphthalene was 500 °C, differing from other contaminants including Zn, Cu, and fluoranthene (typically 700 °C). Time (0.5–8 h) had no significant effect like temperature on these contaminants during calcination. Considering the remediation cycle requirements and economic costs of engineering, we suggested that the optimal calcination condition for Zn, Cu, naphthalene, and fluoranthene was at 700 °C for 0.5 h, and the corresponding stabilization or removal efficiency values were 96.95%, 98.41%, 98.49%, and 98.04%, respectively. Overall, calcination as a remedial strategy exhibits a bright future for practical applications in the simultaneous stabilization of heavy metals and PAH removal from soils. This work also provides new perspectives on further remediation of co-contaminated soils with heavy metals and organic compounds. 

## Figures and Tables

**Table 1:** Physicochemical properties of polycyclic aromatic hydrocarbons (PAHs).

PAHs	Molecular Weight (g/mol)	Boiling Point (°C)	Melting Point (°C)	LogK_ow_	Water Solubility (mg/L)	Molecular Structure
Naphthalene	128.18	217.9	80.5	3.37	31.00	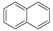
Fluoranthene	202.25	384	111	4.58	0.26	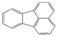

Note: K_ow_ is the octanol–water partition coefficient.

**Table 2:** The stabilization efficiency (%) of zinc in soils by calcination technique.

Temperature (°C)	Time (h)				
	0.5	1	2	4	8
300	58.45 ± 6.85 c	63.40 ± 9.27 c	49.98 ± 5.78 c	37.78 ± 2.00 c	43.15 ± 2.61 c
400	83.50 ± 3.73 b	84.18 ± 2.98 b	80.78 ± 3.69 b	85.64 ± 1.58 b	84.74 ± 4.18 b
500	93.27 ± 0.69 a	93.69 ± 5.86 a	92.72 ± 0.42 a	93.12 ± 0.39 a	94.22 ± 0.19 a
600	94.57 ± 0.19 a	94.98 ± 0.43 a	95.15 ± 0.42 a	95.22 ± 0.73 a	95.97 ± 0.70 a
700	96.95 ± 1.75 a	96.72 ± 0.46 a	95.77 ± 1.35 a	95.96 ± 0.29 a	96.09 ± 0.66 a

Different letters indicate significant difference (*p* ≤ 0.05) between treatments of different calcination temperatures for the same time.

**Table 3:** The stabilization efficiency (%) of copper in soils by calcination technique.

Temperature (°C)	Time (h)				
	0.5	1	2	4	8
300	93.25 ± 1.61 c	94.04 ± 2.07 b	92.37 ± 0.82 c	90.61 ± 0.40 e	93.00 ± 0.59 c
400	96.18 ± 0.39 b	96.39 ± 0.38 a	96.34 ± 0.24 bc	96.41 ± 0.30 d	97.05 ± 0.60 ab
500	97.40 ± 0.27 ab	97.44 ± 0.22 a	97.16 ± 0.19 b	97.42 ± 0.15 b	97.63 ± 0.03 a
600	97.90 ± 0.07 a	97.92 ± 0.20 a	98.06 ± 0.20 a	98.25 ± 0.11 a	98.27 ± 0.03 a
700	98.41 ± 0.37 a	97.68 ± 0.49 a	97.05 ± 0.22 b	96.92 ± 0.08 c	96.90 ± 0.13 b

Different letters indicate significant difference (*p* ≤ 0.05) between treatments of different calcination temperatures for the same time.

**Table 4:** The removal efficiency (%) of naphthalene in soils by calcination technique.

Temperature (°C)	Time (h)				
	0.5	1	2	4	8
300	99.30 ± 0.03 c	99.12 ± 0.03 b	99.57 ± 0.10 b	98.55 ± 0.13 c	98.63 ± 0.12 b
400	97.72 ± 0.08 e	97.62 ± 0.14 d	99.15 ± 0.13 c	98.51 ± 0.06 c	98.03 ± 0.10 c
500	99.94 ± 0.05 a	99.95 ± 0.05 a	99.91 ± 0.02 a	99.41 ± 0.05 a	99.41 ± 0.10 a
600	99.50 ± 0.04 b	98.51 ± 0.09 c	97.17 ± 0.16 d	97.98 ± 0.07 d	97.46 ± 0.08 d
700	98.49 ± 0.07 d	98.41 ± 0.14 c	99.23 ± 0.11 c	99.15 ± 0.13 b	98.57 ± 0.04 b

Different letters indicate significant difference (*p* ≤ 0.05) between treatments of different calcination temperatures for the same time.

**Table 5:** The removal efficiency (%) of fluoranthene in soils by calcination technique.

Temperature (°C)	Time (h)				
	0.5	1	2	4	8
300	94.75 ± 0.05 e	95.95 ± 0.05 c	96.17 ± 0.03 c	94.70 ± 0.00 e	95.63 ± 0.03 e
400	96.34 ± 0.04 d	96.74 ± 0.04 b	95.57 ± 0.03 c	96.03 ± 0.03 d	96.27 ± 0.03 d
500	96.72 ± 0.02 c	96.55 ± 0.43 b	97.93 ± 0.78 a	97.88 ± 0.02 b	97.71 ± 0.01 c
600	97.27 ± 0.03 b	96.33 ± 0.03 bc	97.12 ± 0.02 b	97.19 ± 0.01 c	98.08 ± 0.02 b
700	98.04 ± 0.03 a	97.34 ± 0.04 a	98.29 ± 0.01 a	98.92 ± 0.02 a	98.63 ± 0.03 a

Different letters indicate significant difference (*p* ≤ 0.05) between treatments of different calcination temperatures for the same time.
